# Enantioselective metabolism of primaquine by human CYP2D6

**DOI:** 10.1186/1475-2875-13-507

**Published:** 2014-12-17

**Authors:** Pius S Fasinu, Babu L Tekwani, NP Dhammika Nanayakkara, Bharathi Avula, HMT Bandara Herath, Yan-Hong Wang, Vijender R Adelli, Mahmoud A Elsohly, Shabana I Khan, Ikhlas A Khan, Brandon S Pybus, Sean R Marcsisin, Gregory A Reichard, James D McChesney, Larry A Walker

**Affiliations:** The National Center for Natural Products Research, University of Mississippi, University, Kragujevac, MS 38677 USA; BioMolecular Sciences, University of Mississippi, University, Kragujevac, MS 38677 USA; Pharmaceutical Sciences and Drug Delivery, School of Pharmacy, University of Mississippi, University, Kragujevac, MS 38677 USA; ElSohly Laboratories, Inc, 5 Industrial Park Dr, Oxford, MS38655 USA; Military Malaria Research Program, Division of Experimental Therapeutics, Walter Reed Army Institute of Research, Silver Spring, MD 20910 USA; Ironstone Separations, Inc, Etta, MS 38627 USA

**Keywords:** Primaquine enantiomers, CYP2D6, Metabolism, Ring-hydroxylation, Quinone-imines, Orthoquinone

## Abstract

**Background:**

Primaquine, currently the only approved drug for the treatment and radical cure of *Plasmodium vivax* malaria, is still used as a racemic mixture. Clinical use of primaquine has been limited due to haemolytic toxicity in individuals with genetic deficiency in glucose-6-phosphate dehydrogenase. Earlier studies have linked its therapeutic effects to CYP2D6-generated metabolites. The aim of the current study was to investigate the differential generation of the CYP2D6 metabolites by racemic primaquine and its individual enantiomers.

**Methods:**

Stable isotope ^13^C-labelled primaquine and its two enantiomers were incubated with recombinant cytochrome-P450 supersomes containing CYP2D6 under optimized conditions. Metabolite identification and time-point quantitative analysis were performed using LC-MS/MS. UHPLC retention time, twin peaks with a mass difference of 6, MS-MS fragmentation pattern, and relative peak area with respect to parent compound were used for phenotyping and quantitative analysis of metabolites.

**Results:**

The rate of metabolism of (+)-(*S*)-primaquine was significantly higher (50% depletion of 20 μM in 120 min) compared to (−)-(*R*)-primaquine (30% depletion) when incubated with CYP2D6. The estimated *V*_*max*_ (μmol/min/mg) were 0.75, 0.98 and 0.42, with *K*_*m*_ (μM) of 24.2, 33.1 and 21.6 for (±)-primaquine, (+)-primaquine and (−)-primaquine, respectively. Three stable mono-hydroxylated metabolites, namely, 2-, 3- and 4-hydroxyprimaquine (2-OH-PQ, 3-OH-PQ, and 4-OH-PQ), were identified and quantified. 2-OH-PQ was preferentially formed from (+)-primaquine in a ratio of 4:1 compared to (−)-primaquine. The racemic (±)-primaquine showed a pattern similar to the (−)-primaquine; 2-OH-PQ accounted for about 15–17% of total CYP2D6-mediated conversion of (+)-primaquine. In contrast, 4-OH-PQ was preferentially formed with (−)-primaquine (5:1), accounting for 22% of the total (−)-primaquine conversion. 3-OH-PQ was generated from both enantiomers and racemate. 5-hydroxyprimaquine was unstable. Its orthoquinone degradation product (twice as abundant in (+)-primaquine compared to (−)-primaquine) was identified and accounted for 18–20% of the CYP2D6-mediated conversion of (+)-primaquine. Other minor metabolites included dihydroxyprimaquine species, two quinone-imine products of dihydroxylated primaquine, and a primaquine terminal alcohol with variable generation from the individual enantiomers.

**Conclusion:**

The metabolism of primaquine by human CYP2D6 and the generation of its metabolites display enantio-selectivity regarding formation of hydroxylated product profiles. This may partly explain differential pharmacologic and toxicologic properties of primaquine enantiomers.

## Background

Primaquine, a prototype 8-aminoquinoline, the only licensed option to treat the relapsing liver stages (hypnozoites) of *Plasmodium vivax*, is also used for the prophylaxis of all forms of human malaria [1–4]. Due to its activity against mature, infective *Plasmodium falciparum* gametocytes, this drug can be employed clinically as a gametocytocide for blocking transmission in *P. falciparum* malaria. In areas of emerging drug resistance, primaquine has shown effectiveness against and in the prevention of the spread of artemisinin-resistant *P. falciparum* strains [5]. Since its introduction into the market in the 1950s, primaquine has been documented to trigger haemolysis in individuals with a genetic deficiency in glucose-6-phosphate dehydrogenase (G6PD) [3, 4]. Thus, despite the unique therapeutic indications of primaquine, the widespread prevalence of G6PD deficiency across populations in malaria-endemic areas has limited its clinical use [6, 7].

In 1962, Tarlov and co-workers suggested that a metabolite of primaquine, rather than primaquine itself, might be responsible for the haematotoxicity of primaquine [8]. This was based on the observed delay in anticatalase activity in an individual dosed with the drug; it was postulated that redox-cycling of the metabolite 6-demethyl-5-hydroxyprimaquine and its corresponding orthoquinone caused oxidative stress [8]. Even though positive identification in metabolic mixtures could be made only during the last decade [9] due to the high reactivity of the metabolites and limitations of available analytical methods, a number of *in vitro* studies have shown that 5-hydroxyprimaquine and its oxidative products caused haematotoxicity [10–13]. Carboxyprimaquine, which has been identified as the major circulating metabolite in several species, including man, after oral administration of primaquine [14] has been found to be non-toxic [11] and inactive [15]. An earlier *in vitro* study suggested that multiple CYP isoforms (CYP2E1, CYP2B6, CYP1A2, CYP2D6, and CYP3A4) variably contributed to the haemotoxic response of primaquine [16]. Monoamine oxidase (MAO) has been shown as responsible for the formation of carboxyprimaquine [17], whereas CYP enzymes, especially CYP2D6, were found to produce several ring-hydroxylated primaquine metabolites [18].

Recent work using CYP2D knockout and humanized CYP2D6 mice, highlighted the dependence of the efficacy of primaquine and other 8-aminoquinolines (8AQ) on CYP2D metabolism in a rodent causal prophylaxis model [19, 20]. The human relevance of this finding was substantiated by the observed treatment failures of primaquine in humans with *P. vivax* malaria who are deficient in CYP2D6 enzyme activity which suggested that CYP2D6 is required for the generation of the active metabolites [21].

Primaquine is a chiral drug and is currently used as a racemic mixture, approximating a 50:50 ratio of (+)-(*S*)- and (−)-(*R*)-enantiomers. Previously, differential activity, toxicity and pharmacokinetic profiles for the individual enantiomers of primaquine have been shown [22–24]. (+)-(*S*)-primaquine showed better causal prophylactic and blood schizonticidal activities in *Plasmodium berghei* mouse malaria models and higher propensity to cause haematotoxicity in a non-obese diabetic/severe combined immunodeficiency (NOD/SCID) mouse model engrafted with G6PD-deficient human erythrocytes and beagle dogs [23]. However, in rhesus monkeys, the results [24] were in agreement with the report by Schmidt et al. [22] that both enantiomers had equivalent radical curative activity against *Plasmodium cynomolgi*. The stereo-selectivity in metabolite generation and other pharmacokinetic behavior of chiral antimalarial drugs has been known to lead to major differences in pharmacodynamic properties of individual enantiomers [25]. Similarly, the enantioselective pharmacologic and toxicologic properties of primaquine may be attributed to differential pharmacokinetic profiles of the two enantiomers. Studies with mice and humans have shown that the major serum metabolite, carboxyprimaquine, which amounted to more than 60% of total metabolites was predominantly emanating from (−)-(*R*)-primaquine [26].

Considering the essential contribution of CYP2D6-linked metabolism to the efficacy and toxicity of primaquine, and the generation of multiple quinoline ring-hydroxylated metabolites on incubation of racemic primaquine with CYP2D6, it was hypothesized that observed variation in therapeutic response of the two primaquine enantiomers may be attributed to enantio-selective CYP2D6-mediated ring hydroxylation of primaquine. Challengingly, the low quantities and the highly reactive nature of the ring-hydroxylated primaquine metabolites pose major problems regarding phenotyping and quantification of these metabolites. Recently this challenge has been addressed by the application of 50:50 mixture of ^13^C-stable isotope labelled (C_6_) and normal ^12^C- primaquine followed by analysis with liquid chromatography–mass spectrometry (LC-MS/MS) [9].

For the identification of metabolites, 2-, 3-, 4-, and 5-hydroxyprimaquine (2-OH-PQ, 3-OH-PQ, 4-OH-PQ and 5-OH-PQ) and 8-*N*-hydroxyprimaquine were prepared as reference standards. Attempts at preparation of 7-hydroxyprimaquine have been unsuccessful to date. 5-OH-PQ spontaneously underwent oxidation yielding the orthoquinone product.

Comparison of ultra-high performance liquid chromatography (UHPLC) retention times (RT) of twin mass peaks with difference of 6 (originating from ^13^C_6_- primaquine /^12^C- primaquine) with those of reference standards, MS-MS fragmentation patterns, and relative peak area with respect to parent compound were used for phenotyping and semi-quantitative analysis of the metabolites.

## Methods

### Synthetic chemicals

^13^C(6)-labelled primaquine (racemic, and the (+)-(*S*)- and (−)-(*R*)- enantiomers) [27] and primaquine alcohol [28] were synthesized as previously reported. The identity of the compounds synthesized was confirmed by spectral infra-red (IR), nuclear magnetic resonance (NMR) and high-resolution MS and physical data in comparison with published values. 4-OH-PQ and 5-OH-PQ were prepared using the previously reported procedures [29, 30]. The latter underwent rapid spontaneous oxidation. Time-lapse LC-MS analysis indicated formation of quinone-imine, which underwent demethylation and isomerization yielding a stable 5,6-orthoquinone analog. The methods for synthesis of other analogs, which were used as standards for phenotyping and quantification of the primaquine metabolites, are described below.

### Synthesis of 2-hydroxyprimaquine diphosphate

#### 2-benzyloxy-6-methoxy-8-(1-methyl-4-phthalimidobutylamino)quinoline

A mixture of 2-benzyloxy-6-methoxy-8-nitroquinoline [31] (2.75 g) hydrazine hydrate (3 ml) and Raney Ni (1.5 g) in ethanol (75 ml) was refluxed for four hours. The catalyst was removed by filtration through celite and the filtrate was evaporated. The residue was partitioned between CH_2_Cl_2_ and water and the organic layer was dried with sodium sulphate and evaporated to give 8-amino-2-benzyloxy-6-methoxyquinoline (2.3 g). To a stirred mixture of this product (2.3 g, 7.8 mm) and 2-oxo-5-phthalimido pentane (2.0 g, 8.6 mm) in glacial acetic acid (30 ml), sodium borohydride was added portion-wise while maintaining the temperature below 30°C until reaction was complete evidenced by thin layer chromatography (TLC). The reaction mixture was poured onto ice and basified with aqueous sodium hydroxide (50%). The product was separated by filtration and purified by chromatography on silica gel with hexanes:ethyl acetate 85:15 to give 2-benzyloxy-6-methoxy-8-(1-methyl-4-phthalimidobutylamino)quinoline as a yellow crystalline solid (3.3 g)^. 1^H NMR δ (CDCl_3_): 1.27 (3H, d, J = 6.0 Hz), 1.60–1.90 (4H, m), 3.62 (1H, m), 3.73 (2H, t, J = 6.8 Hz), 3.84 (3H, s), 5.46 (2H, s), 5.54 (1H, d, J = 6.8 Hz), 6.31 (2H, s), 6.91 (1H, d, J = 8.8 Hz), 7.23 (1H, t, J = 7.6 Hz), 7.38 (2H, t, 7.6 Hz), 7.51 (2H, d, J = 7.6 Hz), 7.66 (2H, m) 7.78 (2H, m), 7.81 (1H, d, 8.8 Hz); HRESIMS [M + H]^+^*m/z* 496.2232 (calculated for (C_30_H_29_N_3_O_4_ + H)^+^ 496.2236).

#### 2-hydroxyprimaquine diphosphate

A mixture of 2-benzyloxy-6-methoxy-8-(1-methyl-4-phthalimidobutylamino)quinoline (2.0 g) and hydrazine hydrate (1.5 ml) in ethanol (50 ml) was refluxed for four hours. The reaction mixture was cooled and the white precipitate was separated by filtration. The filtrate was evaporated and the gummy residue was dissolved in CH_2_Cl_2_ and washed (× 2) aqueous potassium hydroxide (10%), water, dried over Na_2_SO_4_ and evaporated to give *N*^4^-(2-(benzyloxy)-6-methoxyquinolin-8-yl)pentane-1,4-diamine. This product was dissolved in ethanol (50 ml) and refluxed with hydrazine hydrate (2 ml) and Pd/C (10%, 200 mg) for two hours. The catalyst was removed by filtration and the filtrate was evaporated under reduced pressure. The resulting yellow solid was dissolved in ethanol (10 ml) and H_3_PO_4_ (85%, 2 ml) was added drop-wise under stirring. The supernatant was removed by decantation and the gummy deposit was washed with ethanol and crystallized from water ethanol to give 2-OH-PQ diphosphate (1.6 g). ^1^H NMR δ (CDCl_3_): 0.93 (3H, d, J = 6.0 Hz), 1.28 (1H, m), 1.40 (1H, m) 1.42–1.58 (2H, m), 2.77 (2H, t, J = 7.6 Hz), 3.08 (1H, m), 3.44 (3H, s), 5.76 (1H, brs), 5.84 (1H, brs), 6.06 (H, d, J = 9.2 Hz), 7.12 (1H, d, 9.2 Hz); HRESIMS [M + H]^+^*m/z* 276.1710 (calculated for (C_15_H_21_N_3_O_2_ + H)^+^ 276.1712).

### Synthesis of 3-hydroxyprimaquine diphosphate

#### 3-hydroxy-6-methoxy-8-nitroquinoline

A mixture of 3-bromo-6-methoxy-8-nitroquinoline [32], (2 g, 7 mm), Pd_2_dba_3_ (130 mg, 0.14 mm), 2-di-tert-butylphosphino-2′,4′,6′-triisopropyibiphenyl (140 mg, 0.33 mm), and KOH (1.2 g, 21 mm) in water:dioxane (1:1, 20 ml) was heated at 95°C for two hours under nitrogen atmosphere. The reaction mixture was poured onto ice, acidified with hydrochloric acid and filtered. The solid obtained was purified by column chromatography on silica gel with hexanes:ethyl acetate 7:3 as the eluent to give a yellow crystalline solid (1.36 g). ^1^H NMR δ (CDCl_3_/CD_3_OD): 3.79 (3H, s), 7.01 (1H, d, J = 2.8 Hz), 7.26 (1H, d, J = 2.8 Hz) 7.35 (H, d, J = 2.4 Hz), 8.35 (1H, d, 2.4 Hz); HRESIMS [M + H]^+^*m/z* 221.0572 (calculated for (C_10_H_9_N_2_O_4_ + H)^+^ 221.0562).

#### 3-benzyloxy-6-methoxy-8-nitroquinoline

A mixture of 3-hydroxy-6-methoxy-8-nitroquinoline (1.35 g, 6.1.mm), benzyl bromide (1.71 g, 10 mm), and Cs_2_CO_3_ (3.25 gm, 10 mm) in DMF (15 ml) was stirred at 65°C for six hours. The reaction mixture was poured into cold water and filtered. The crude product was chromatographed over silica gel and elution with hexanes:ethyl acetate 9:1 gave 3-benzyloxy-6-methoxy-8-nitroquinoline (1.8 gm). ^1^H NMR δ (CDCl_3_): 3.81 (3H, s), 5.35 (2H, s), 6.49 (1H, d, J = 2.4 Hz), 6.68 (1H, d, J = 2.4 Hz) 7.28 (1H, t, J = 6.8 Hz), 7.35 (2H, t, J = 6.8 Hz), 7.48 (2H, d, J = 6.8 Hz), 8.09 (1H, d, 2.4 Hz), 8.75 (1H, d, 2.4 Hz); HRESIMS [M + H]^+^*m/z* 311.1050 (calculated for (C_17_H_15_N_2_O_4_ + H)^+^ 311.1032).

#### 3-benzyloxy-6-methoxy-8-(1-methyl-4-phthalimidobutylamino)quinoline

A mixture of 3-benzyloxy-6-methoxy-8-nitroquinoline (1.8 gm), Raney-Ni (1 g) and hydrazine hydrate (2 ml) in ethanol (30 ml) was refluxed for four hours. The catalyst was removed by filtration through a celite plug and the filtrate was evaporated under reduced pressure. The gummy residue was partitioned between water and CH_2_Cl_2_ and the organic layer was dried and evaporated to yield 8-amino-3-benzyloxy-6-methoxyquinoline (1.6 g, 5.7 mm). This product and 2-oxo-5-phthalimido pentane (1.5 g, 6.8 mm) were coupled in glacial acetic acid (15 ml) as described above for 2-benzyloxy analog to afford 3-benzyloxy-6-methoxy-8-(1-methyl-4-phthalimidobutylamino)quinoline as a yellow crystalline solid (1.9 g). ^1^H NMR δ (CDCl_3_): 1.27 (3H, d, J = 6.0 Hz), 1.60–1.90 (4H, m), 3.64 (1H, m), 3.72 (2H t, J = 7.6 Hz), 3.86 (3H, s), 5.15 (2H, s), 5.87 (1H, d, J = 8.4 Hz), 6.15 (1H, d, J = 2.4 Hz), 6.22 (1H, d, J =2.4 Hz), 7.26 (1H, d, J =2.4 Hz), 7.35 (1H, t, J = 6.8 Hz), 7.41 (2H, t, 7.2 Hz), 7.47 (2H, d, J = 7.2 Hz), 7.68 (2H, m) 7.81 (2H, m), 8.33 (1H, d, 2.4 Hz); HRESIMS [M + H]^+^*m/z* 496.2229 (calculated for (C_30_H_29_N_3_O_4_ + H)^+^ 496.2236).

#### 3-hydroxyprimaquine diphosphate

The phthalimide protecting group of 3-benzyloxy-6-methoxy-8-(1-methyl-4-phthalimidobutylamino)quinoline (1.8 g) was removed by reacting with hydrazine hydrate (1 ml) in ethanol (40 ml) as described for 2-benzyloxy analog to give *N*^4^-(3-(benzyloxy)-6-methoxyquinolin-8-yl)pentane-1,4-diamine as a yellow gum. The benzyl group was removed as described above by refluxing for two hours in ethanol (30 ml) in the presence of hydrazine hydrate (1 ml) and Pd/C (10%, 200 mg). The reaction was worked up and 3-OH-PQ was crystallized as diphosphate (1.4 g). ^1^H NMR δ (CDCl_3_): 1.10 (3H, d, J = 6.0 Hz), 1.30–1.65 (4H, m), 2.81 (2H, t, J = 7.2 Hz), 3.45 (1H, m), 3.65 (3H, s), 6.28 (1H, brs), 7.21 (H, d, J = 2.4 Hz), 7.97 (1H, d, 2.4 Hz); HRESIMS [M + H]^+^*m/z* 276.1728 (calculated for (C_15_H_21_N_3_O_2_ + H)^+^ 276.1712).

### Other chemicals and reagents

Nicotinamide adenine dinucleotide phosphate, reduced form (NADPH), glucose-6-phosphate (G6P), G6PD and magnesium chloride (MgCl_2_) were purchased from Sigma-Aldrich (St Louis, MO, USA). HPLC-grade acetonitrile and methanol were purchased from Fisher Scientific (Fair Lawn, NJ, USA). Water for the HPLC mobile phase was purified in a Milli-Q system (Millipore, Bedford, MA, USA). Baculovirus-insect cell expressed recombinant cytochrome-P450 supersomes containing CYP2D6 (1 nmole CYP per mL) were purchased from (BD Biosciences, Billerica, MA, USA) and stored at −80°C until used.

### Primaquine/CYP2D6 incubation

The *in vitro* primaquine metabolism reactions were set up in a clear 96-well plate. Thawed suspensions of the supersomes were diluted with potassium phosphate buffer (50 mM; pH =7.4) and aliquots were dispensed in a clear 96-well plate. Primaquine (racemate or the appropriate enantiomer, 50:50 mixture of ^12^C and ^13^C-labeled) was added and the mixture pre-incubated at 37°C for 10 min. Metabolic reactions were initiated by adding the NADPH-regenerating solution containing magnesium chloride, G6P and G6PD. The final components of the incubations were: reduced NADP (1 mM), G6P (5 mM), MgCl_2_ (5 mM), G6PD (1 U/mL), recombinant CYP2D6 (0.5 mg/mL) and primaquine (varying concentrations). Metabolic reactions were terminated at predetermined time-points through the addition of equal volume of ice-cold methanol containing 0.5 μg/mL 6-D_3_-methoxyprimaquine as internal standard. The mixtures were kept on ice for one hour and then centrifuged (14,000 rpm, −4 degree centigrade, 20 min). Clear supernatants were kept for LC-MS analysis. All incubations were performed in duplicate for intra-day agreement and repeated on separate days for inter-day comparisons. Control incubations included: a) those with primaquine but without the supersomes; b) those with supersomes and primaquine but without the start solution; and, c) those without the primaquine. There was no organic solvent in the incubation mixtures as the contents were water-soluble. After the initial determination of the kinetic parameters of primaquine and its enantiomers, the probe primaquine concentrations for the metabolite identification and quantification were less than the determined *Km* value.

### Detection, identification and quantification of metabolites

Liquid chromatography – mass spectrometry (LC-MS) method for simultaneous analysis of primaquine and its metabolites as reported earlier was employed in this study [9]. Total separation and elution of the analytes were achieved within 10 min retention time, using the ACQUITY UHPLC™, BEH Shield RP18 column (100 mm × 2.1 mm I.D., 1.7 mm) equipped with an LC-18 guard column (Vanguard 2.1 × 5 mm, Waters Corp, Milford, MA, USA) on an ACQUITY UHPLC system (Waters Corp, Milford, MA, USA) to which a conditioned auto-sampler (at 20°C) was attached. The mobile phase, consisting of water with 0.05% formic acid (A) and acetonitrile with 0.05% formic acid (B), was applied at a flow rate of 0.25 ml/min in the following linear gradient elution: 0 min, 90% A:10% B in next 5 min to 63% A:37% B, then for 3 min 37% A:63% B and to 100% B in next 2 min. Each run was followed by a 3-min wash with 100% B and an equilibration period of 3.5 min with 90% A/10% B. Ten μL of each sample were injected, and peaks assigned with respect to the mass of the compounds and comparison of the retention times.

Metabolites in the accurate mass data were found using the Metabolynx® software. The data were searched using predicted metabolite mass, mass defects, isotope, and fragmentation patterns. Each sample was subjected to data acquisition in full scan and data-dependent positive MS/MS, targeted MS/MS (ESI positive ionization mode) and high-resolution MS (HRMS) modes using the Waters ACQUITY^TM^ XEVO QTOF Mass Spectrometer (Waters Corporation, Manchester, UK) connected to the UHPLC system via an electrospray ionization (ESI) interface. Identification of each metabolite was assisted by its HRMS data, which were used to calculate their elemental compositions. The full scan mass data were screened and filtered using Waters MetaboLynx XS software. The qualitative metabolite identification was performed using this software package.

### Data analysis

After suitable calibrations of the substrates and the synthetic metabolites, the initial rate of primaquine (racemate and its enantiomers) metabolism was profiled against the concentration incubated using the SigmaPlot Enzyme Kinetic Software, Module 13.0 (Systat Software Inc, Chicago, IL, USA). The pattern of metabolism was characterized through the Michaelis-Menten plot (N = 4; S.D. < 0.05 in all cases, R2 ≥ 0.9) from which the kinetic parameters were determined. Identified metabolites were quantified and profiled against time.

## Results

### Comparative kinetics for metabolism of primaquine enantiomers

**Figure 1:**
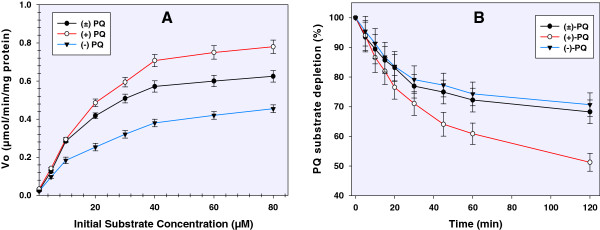
**Concentration- and time-dependent metabolism of primaquine and its enantiomers. (A)** Steady state kinetics of primaquine and its enantiomers – a profile of the initial rate of substrate depletion (*V*_*o*_) against concentration and **(B)** comparative depletion of primaquine and its enantiomers from human CYP2D6 incubation over 2 hr. Each point represents mean values ± S.D. (n = 4).

Varying concentrations of primaquine and its enantiomers were subjected to human CYP2D6-catalyzed metabolism. The initial velocity of metabolism (*V*_*o*_) was profiled against substrate concentration yielding a Michaelis-Menten-type curve (Figure 1A). The rate of metabolism of (+)-primaquine was significantly higher (50% depletion of 20 mM primaquine in 120 min) compared to (−)-primaquine (30% depletion). The rate of metabolism of racemic primaquine was similar to (−)-primaquine. The estimated *Vmax* (μmol/min/mg) are 0.87, 0.75 and 0.42 for (±)-primaquine, (+)-primaquine and (−)-primaquine, respectively. The estimated Michaelis-Menten constants (*K*_*m*_) (μM) for (±)-primaquine, (+)-primaquine and (−)-primaquine were 24.2, 33.1 and 21.6, respectively. The results in Figure 1A illustrate that (−)-primaquine saturated CYP2D6 earlier than (+)-primaquine or the racemic mixture. Additionally, the Michaelis-Menten kinetic analyses indicated that (−)-primaquine has a higher affinity for CYP2D6 as compared to (+)-primaquine.

The depletion of primaquine substrates from the initial concentration (*C*_*o*_) *vs* time is presented in Figure 1B. After two hours, the amount of (+)-primaquine depleted is more than 50% greater than the (−)-primaquine consumed. These profiles suggest further that the CYP2D6 metabolism of primaquine is stereo-selective and more favourable towards (+)-primaquine.

### Metabolite identification

**Figure 2:**
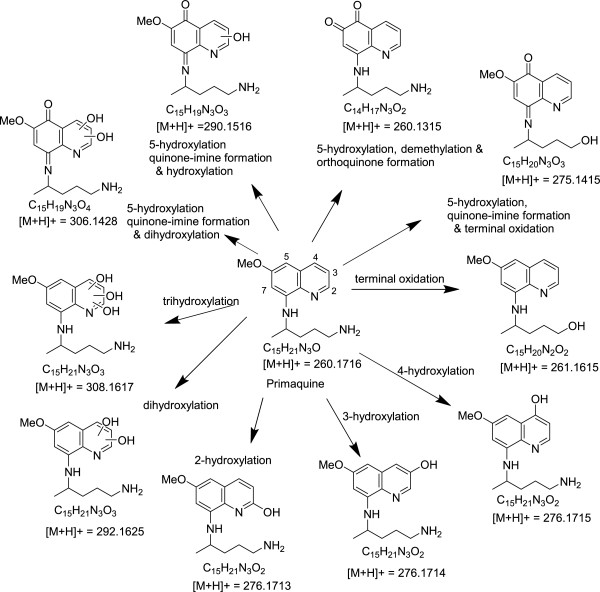
**Putative metabolic pathways of primaquine with human CYP2D6.**

Following the incubation of primaquine (racemate and individual enantiomers) *in vitro* with recombinant human CYP2D6, several metabolites were identified. Quantitatively these metabolites were formed at varying levels with (+) and (−)-primaquine. Table 1 provides a list of the identified metabolites and their comparative abundance in the enantiomers. Formation of some of these metabolites has been reported with the racemic primaquine [18]. Three stable mono-hydroxylated metabolites namely, 2-OH-PQ, 3-OH-PQ and 4-OH-PQ were identified, confirmed with analytical standards and quantified. 5,6-orthoquinone analog (*m/z* 260), the product of the spontaneous transformation of 5-OH-PQ was identified, as one of the most abundant primaquine metabolite formed with CYP2D6. Smaller amounts of two other metabolites corresponding to dihydroxylated metabolites and identified as respective quinone-imines or orthoquiones (*m/z* 290) were also identified. The two dihydroxymetabolites exhibited different retention times (3.42 and 4.5 min), probably due to hydroxylation at different positions on the quinoline ring. Trace amounts of primaquine terminal alcohol were also detected. Figure 2 shows suggested pathways for metabolism of primaquine by human CYP2D6.

**Table 1:** **Identified metabolites from CYP2D6-mediated metabolism of primaquine and its enantiomers**

Peak [M+H] ^+^	RT	Formula	Remarks
260.13	1.5	C_14_H_17_N_3_O_2_	Oxidized product of 5-OH-PQ, preferentially generated from the (+)-primaquine (twice as much compared to (−)-primaquine)
261.16	8.7	C_15_H_20_N_2_O_2_	Oxidative deamination of primaquine and subsequent reduction to primaquine alcohol.
275.14	8.8	C_15_H_18_N_2_O_3_	Possibly terminal amine oxidation, with oxidation and quinone-imine formation on quinoline ring, predominantly formed from (−)-primaquine (2:1)
276.17	2.08	C_15_H_21_N_3_O_2_	Identified as 4-OH-PQ; Formed 5 times more predominantly with (−)-primaquine than (+)-primaquine
276.17	3.64	C_15_H_21_N_3_O_2_	Identified as 2-OH-PQ; Generated in the ratio 4:1 by (+)-primaquine *vs* (−)-primaquine
276.17	4.78	C_15_H_21_N_3_O_2_	Identified as 3-OH-PQ; Generated in the ratio 2:3 by (+)-primaquine and (−)-primaquine
290.15	3.42	C_15_H_20_N_3_O_3_	Putatively identified as dihydroxylated primaquine metabolite converted to quinone-imines, generated in the ratio 3:2 by (−)-primaquine and (+)-primaquine.
290.15	4.56	C_15_H_20_N_3_O_3_	Putatively identified as a dihydroxylated primaquine metabolite converted to quinone-imine; Generated more predominantly with (−)-primaquine, minimally with (±)-primaquine and not detected with (+)-primaquine
292.16	4.59	C_15_H_21_N_3_O_3_	Putatively identified as dihydroxyprimaquine; Generated with (−)-primaquine only
306.14	3.65	C_15_H_20_N_3_O_4_	Putatively identified as trihydroxylated primaquine converted to the quinone-imine; selectively generated with (+)-primaquine
308.16	1.4	C_15_H_21_N_3_O_4_	Putatively identified as trihydroxyprimaquine; detected in trace amount; more prominently generated with (+)-primaquine

### 2-hydroxyprimaquine

**Figure 3:**
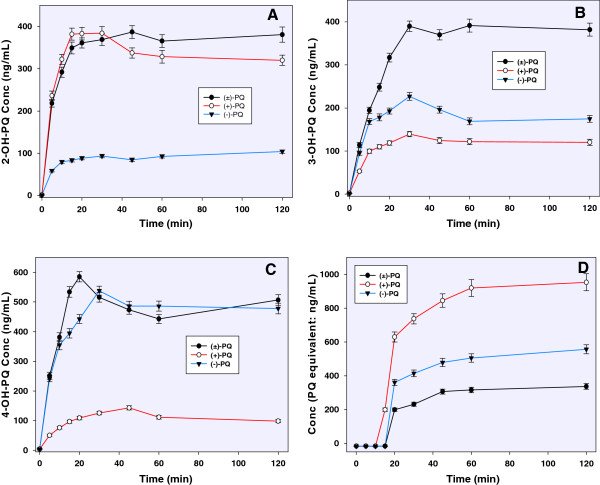
**Comparative kinetics of formation of (A) 2-hydroxyprimaquine; (B) 3-hydroxyprimaquine; (C) 4-hydroxyprimaquine and (D) 5-hydroxyprimaquine (expressed as the orthoquinone, relative to primaquine calibration) on metabolism of primaquine (racemate and individual enantiomers) by human CYP2D6.** Each point represents mean value ± S.D. (n = 4).

The identification of 2-OH-PQ was confirmed through the availability of its synthetic standard. 2-OH-PQ was preferentially formed from (+)-primaquine peaking early within 15 min at about 390 ng/mL compared to the peak of less than 100 ng/mL generated by (−)-primaquine (Figure 3A). The rate of metabolism of the racemic (±)-primaquine to 2-OH-PQ was similar to that of the (+)-primaquine. A small reduction in the level of 2-OH-PQ in was recorded for (+)-primaquine after 30 min, with the level of 2-OH-PQ levelling at about 300 ng/mL, presumably reflecting further metabolism of this species. However, the level of 2-OH-PQ generated from (−)-primaquine peaked at 15 min. The pattern of 2-OH-PQ generation suggests that the amount observed with the racemic (±)-primaquine at two hours approximates the cumulative sum of those generated by the individual enantiomers. Thus, the enantiomers do not compete with each other for their metabolism to 2-OH-PQ with CYP2D6. The 2-OH-PQ accounted for about 15–17% of total CYP2D6-mediated metabolism of (+)-primaquine or (±)-primaquine.

### 3-hydroxyprimaquine

With a retention time of 3.6 min, synthetic 3-OH-PQ was used to confirm the formation of this metabolite with CYP2D6. The level of 3-OH-PQ peaked at 30 min and was formed with 1.5-fold more abundance from (−)-primaquine than with (+)-primaquine, while the levels of 3-OH-PQ generated from (±)-primaquine were apparently the cumulative generation from the individual enantiomers. The 3-OH-PQ also was identified as a stable metabolite (Figure 3B).

### 4-hydroxyprimaquine

The identity of 4-OH-PQ was also confirmed through the synthetic standard with a chromatographic retention time of 2.1 min and *m/z* 276.25. It was preferentially formed with (−)-primaquine, generating five times the quantity observed with (+)-primaquine. It accounts for about 22% of the total metabolism of (−)-primaquine. The pattern of its generation appears to be complementary to the generation of 2-OH-PQ. It peaked at 30 min in (−)-primaquine with slight drop in the quantity generated over the next 90 min (Figure 3C).

### 5-hydroxyprimaquine

5,6-orthoquinone analog (*m/z* 260), the product of the spontaneous transformation of 5-OH-PQ was identified based on its retention time (1.65 min) and fragmentation pattern. It was semi-quantified relative to the parent primaquine calibration. The LC-MS/MS profile of this product as identified in primaquine and CYP2D6 reaction mixture was identical to the oxidation products formed with the synthetic 5-OH-PQ. Based on semi-quantification of this 5,6-orthoquinone product as a marker for 5-OH-PQ, the generation of 5-OH-PQ is estimated to account for 18–20% of the CYP2D6-mediated conversion of (+)-primaquine. The formation of this 5-OH-PQ product from (+)-primaquine was more than twice as abundant as with (−)-primaquine. However, generation of 5-OH-PQ marker product from (±) primaquine was of about half the amount from (−)-primaquine and more than four-fold less than that generated from (+)-primaquine. This observation suggested that at these concentrations, the individual primaquine enantiomers may be competing with each other for metabolism of primaquine through this pathway (Figure 3D).

### Dihydroxylated primaquine metabolite converted to quinone-imines

**Figure 4:**
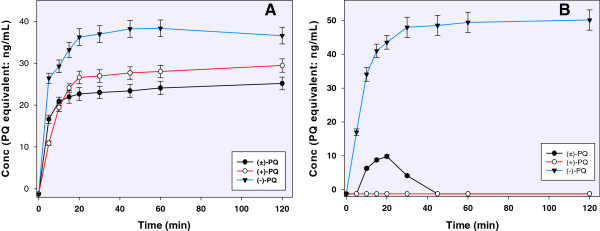
**Semi-quantitation of the human CYP2D6-catalyzed enantio-selective generation of quinone-imine derivative of dihydroxylated primaquine metabolites (A) RT 3.42 min metabolite and (B) RT 4.56 metabolite, identified as quinone-imines (** ***m/z*** **290) degradation product.** Each point shows mean value ± S.D. (n = 4).

The two metabolites, corresponding to dihydroxylated primaquine presumably degraded or further metabolized to quinone-imines or orthoquinones (*m/z* 290), were identified in small quantities. Generation of these metabolites also exhibited differential pattern with racemate primaquine and individual enantiomers (Figure 4). The differential positions of OH on the quinoline ring generated the metabolites with distinctly different retention time. The first of these two quinone-imine products appeared at 3.4 min and was generated in the ratio 3:2, (−)-primaquine *vs* (+)-primaquine (Figure 4A). It was generated in higher levels from the individual enantiomers than from the racemic primaquine, although the level of this metabolite was very low based on semi-quantitation. The second quinone-imine appeared at 4.56 min and was almost exclusively generated from the (−)-primaquine. Only a small amount of this metabolite appeared transiently between 10 and 30 min from (±)-primaquine. No formation of this metabolite was detected from (+)-primaquine. (Figure 4B).

### Dihydroxyprimaquine

**Figure 5:**
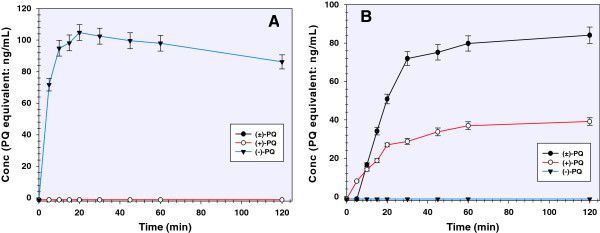
**The human CYP2D6-catalyzed enantio-selective metabolism of primaquine showing the generation of (A) dihydroxyprimaquine (semi-quantified in relation to primaquine calibration) and (B) primaquine alcohol.** Each point shows mean value ± S.D. (n = 4).

Formation of a metabolite, predicted as dihydroxyprimaquine (*m/z* 292) and presumably formed by further hydroxylation of a monohydroxy primaquine, was detected in significant levels (Figure 5A). The exact hydroxylation site in this metabolite could not be established. This metabolite, which was exclusively generated from (−)-primaquine, peaked within 20 min incubation and remained high until end of 120 min of incubation. This metabolite was not detected in the CYP2D6 incubation mixtures with (+)-primaquine or (±)-primaquine.

### Primaquine alcohol

Formation of primaquine alcohol has also been reported earlier [9, 18]. The identity of this metabolite was confirmed based on the similarity of its MS/MS fragmentation with the synthetic primaquine alcohol, having the same retention time and UV spectrum. Primaquine alcohol was exclusively generated from the (+)-primaquine enantiomer (Figure 5B).

## Discussion

The results presented in this paper confirm earlier reports regarding generation of multiple mono-hydroxylated metabolites of primaquine *in vitro* by primary human hepatocytes [16, 17] and CYP2D6 [18, 19]. However, previous studies [16–19] did not provide evidence regarding the specific sites of oxidation on the quinoline ring. The results presented here provide definitive evidence that CYP2D6-mediated oxidation of primaquine occurred at several different positions on the quinoline ring. The identification and quantification of three mono-hydroxylated primaquine metabolites were successfully carried out. Mono-hydroxylated primaquine metabolites were identified as 2-, 3- and 4-OH-PQ by comparing the retention times on LC and MS/MS fragmentation data with those of the synthetic standards. An orthoquinone product of a fourth hydroxyl product was identified as a major CYP2D6 metabolite and is a likely marker for the 5 hydroxylation pathway. Definitive generation of these metabolites from ^13^C(6)-primaquine/^12^C-primaquine (1:1) was confirmed by identification of twin mass peaks for each metabolite. Even though both 2- and 4-OH-PQ can form oxidized products with keto-imine groups, they appear to be stable due to quinolinol-quinolone tautomerism. Further, analysis of the data showed significant quantitative differences in generation of these metabolites from the individual primaquine enantiomers.

This is the first report on differential preferences of human CYP2D6 for individual primaquine enantiomers. The rate of metabolism of (+)-primaquine was about 2 fold higher compared to (−)-primaquine. However, the rate of metabolism of racemic primaquine was similar to (−)-primaquine. This may be due to possible inhibition of the metabolism of one enantiomer by the other. Supposedly, (+)- and (−)-primaquine produce respective (+)- and (−)-metabolites. No isomerization has been detected with PQ and carboxy PQ and has been earlier confirmed with carboxyprimaquine generated in vitro with human hepatocytes (9).

Relative importance of individual hydroxylated primaquine metabolites in efficacy *vs* haemolytic toxicity *vis-à-vis* their preferential generation from the individual primaquine enantiomers are yet to be determined. Preferential formation of the orthoquinone marker of the 5-OH-PQ, reported to be the most reactive primaquine metabolite, may explain the greater haemolytic effect of (+)-primaquine in rodent models [33–36]. However, it has been demonstrated in primates that the two enantiomers share identical radical curative potencies, though the toxicities are qualitatively different [22, 24]. How the two enantiomers compare in efficacy and toxicity in humans is still a matter for further study. It was recently reported that when racemic primaquine is administered in a single dose to human volunteers, the carboxyprimaquine metabolite (quantitatively a major circulating metabolite) is virtually all derived from the (−)-primaquine [Walker *et al*. personal communication].

Understanding the pathways for metabolism of primaquine has been a daunting challenge. Two distinct pathways, the more prominent mono-amine oxidase-catalyzed generation of carboxyprimaquine, the major circulating metabolite, and another mediated through CYP are involved in the metabolism of primaquine. Clear evidence was obtained that specific CYP isoforms could accomplish the conversion of primaquine to methaemoglobin-generating metabolites [8, 35]. Multiple hydroxylated metabolites were formed on *in vitro* incubation of primaquine with human hepatocytes and also CYP2D6 [16, 18]. Hydroxylated metabolites of primaquine are known to be reactive in nature and have been shown to produce haemolytic toxicity [8, 29, 33]. Understanding the precise nature of the hydroxylated metabolites and the determination of their biological efficacy *in vitro* and *in vivo* has been challenging, primarily due to the reactive nature of these metabolites. This challenge has been addressed by the application of sensitive LC-MS/MS analytic methods, stable ^13^C-isotope labelled primaquine and highly reliable metabolite prediction software. Additionally, chemical synthesis of analytical and fully characterized hydroxylated analogs of primaquine, described herein, has provided further confirmation on the nature of several of the hydroxylated metabolites and their precise quantitative analysis.

Recently, CYP2D metabolism was demonstrated to be essential for the causal prophylactic efficacy of primaquine and several other 8-aminoquinoline compounds in mice [19, 20]. The recent report by Bennett *et al*. also demonstrated the requirement for CYP2D6 metabolism for primaquine efficacy in humans [21]. These findings strongly suggest that a CYP2D6-generated metabolite(s) is responsible for the liver stage efficacy of primaquine. Although 5-OH-PQ has previously been suggested as the active metabolite [35–37], it has been difficult to generate conclusive evidence due to the highly reactive nature of this metabolite. The current studies, with chromatographic and mass spectrometric evidence identifying these primary hydroxylated species, afford new tools for probing these questions *in vivo*, and these studies are currently underway.

Differential pharmacological, toxicological, metabolism, and pharmacokinetic profiles of enantiomers of primaquine were recognized many years ago [22], but the findings in several laboratories suggest that these are highly species-dependent [23, 24]. So far it has been difficult to substantially dissociate the efficacy and haemolytic effects of the enantiomers, although some suggestive evidence has been reported [22–24]. The current effort aimed to assess whether the enantiomers differ substantially in their ability to serve as substrates for recombinant human CYP2D6. In contrast to the markedly more prominent metabolism of (−)-primaquine, as compared to (+)-primaquine to carboxyprimaquine [25] (believed to proceed by sequential action of the amine oxidase and aldehyde dehydrogenase), (+)-primaquine was observed to be the preferred substrate for CYP2D6. The overall rate of metabolism of (+)-primaquine by human CYP2D6 was about 1.5-fold faster compared to (−)-primaquine. Taken together, these findings suggest that (−)-primaquine should be less efficacious in humans, by virtue of the lower conversion to 5-OH-PQ marker (5,6 orthoquinone), and greater conversion to the carboxy metabolite, believed to be inactive. However, the ultimate fate of the carboxyprimaquine metabolite, which accumulates to high levels in plasma [14], is not clear. Recent findings suggest that carboxyprimaquine can be further metabolized in human hepatocytes to ring-hydroxylated metabolites [Walker *et al*. personal communication]. The pharmacological or toxicological significance of these, if any, remain to be elucidated. But given the basic structural requirements for CYP2D6 substrates, these are not likely generated via this pathway.

This study presents new evidence regarding different rates and metabolic profiles for the CYP2D6-mediated hydroxylation of primaquine and its enantiomers. It will be important to confirm the biological activities and metabolic profiles of the primaquine enantiomers in humans.

## Conclusion

The metabolism of primaquine by human CYP2D6 and the generation of its metabolites display enantio-selectivity in the hydroxylated product profiles. (+)-Primaquine preferentially generated 2- and 5-OH-PQ while 3- and 4-OH-PQ were predominantly generated from (−)-primaquine. This may partly explain differential pharmacologic and toxicologic properties of primaquine enantiomers. Genetic polymorphism of CYP2D6 in humans leading to the classification of slow and fast metabolizers may be an important consideration in primaquine therapy. The susceptibility of (+)-primaquine to CYP2D6 activity more than (−)-primaquine makes this important if individual enantiomers are administered.

## Abbreviations

G6PD: Glucose-6-phosphate dehydrogenase
NADPH: Nicotinamide adeninine dinucleotide phosphate
2-OH-PQ: 2-hydroxyprimaquine
3-OH-PQ: 3-hydroxyprimaquine
4-OH-PQ: 4-hydroxyprimaquine
5-OH-PQ: 5-hydroxyprimaquine
OH-PQ: Hydroxyprimaquine
MAO: Monoamine oxidase
MgCl_2_: Magnesium chloride
LC-MS/MS: Liquid chromatography-tandem mass spectrometry
UHPLC: Ultra-high performance liquid chromatography
IR: Infra-red
NMR: Nuclear magnetic resonance
TLC: Thin layer chromatography
KOH: Potassium hydroxide
HCl: Hydrochloric acid
RT: Retention time.
